# Conservative Gynecology

**Published:** 1898-06

**Authors:** 


### *EDITORIAL.*

---

The office of The Journal is 311 and 312 Fitten Building.

Address all communications, and make all remittances payable to The Atlanta Medical and Surgical Journal, Atlanta, Ga.

Reprints of articles will be furnished, when desired, at a small cost. Requests for the same should always be made *on the manuscript.*

---

### CONSERVATIVE GYNECOLOGY.

Only a few years ago the medical press was largely interested in what it styled conservative gynecology and vigorous articles were written endeavoring to show that women were often subjected to needless operations, or when operations were performed tissues were often sacrificed which could be saved, and the normal functions of organs often destroyed which could be preserved. A sufficiency of time has now elapsed for us to begin to view this subject from a retrospective standpoint and to judge from results as to whether we were then directing our energies in the right channels or whether many operators of to-day who remove only such tissues as appear macroscopically pathological, are coming closer to the true plan of treatment, viz.: the complete restoration to health with a preservation of functions. These writers were inclined to divide the gynecologists into two classes, the first comprising those who believed that when ovaries and tubes were sufficiently diseased to justify an exposure by operative measures, they were also pathologic enough to warrant removal, or if there be distinct disease of both tubes and ovaries there was no need to leave a uterus which in all likelihood, under microscopic examination, would prove markedly diseased, and would become an organ without function exposed to pathological invasion and liable to undergo malignant degeneration, in fact, only a menace to health or longevity. This class was styled the radicalists, while the second, which will now be described, were self-styled conservatives, including those who often made exploring operations, and on finding extensive adhesions would close the abdomen without removing anything, or on finding adhesions easily broken up would do this either by abdominal or vaginal route, or on exposing an ovary, if any part of its tissue appeared healthy, it was left, or the removal of one organ alone in order that menstruation and often ovulation be preserved, small cysts of the ovaries drained, tubes that were disturbed were drained through the vagina, the uterus not removed except where markedly diseased. So often has it become necessary to subject women who have been in the hands of the extremists in this field to second operations before any relief is afforded their sufferings, that viewed from the present results the tendency seems to point rather toward a medium between these extremes. The leaving of a piece of a pathological ovary has never seemed logical, as it usually becomes imbedded in a mass of adhesions produced by the diseased conditions, and the trauma necessary to remove the part which appears diseased renders it in a great measure functionless and capable of producing decided nervous disturbances. Hence, these, when sufficiently diseased to require removal of part, would lead to better results if the entire ovary was removed. But in such cases as present a decided disuse of one side we can occasionally leave the other ovary, provided it be in a healthy condition, and thus preserve the function of the generative organs. The uterus too can often be saved when both tubes and ovaries are removed in order that the support for the abdominal viscera be preserved. If, however, there be a history pointing toward malignancy it becomes the most conservative method to remove this along with the adnexæ. Hysterectomies have been so much simplified that it is but little more difficult to remove the uterus now than the appendages alone, and some operators are inclined to be a little extreme in this direction.

The nervous disturbances which are attributed to a superinduced menopause open an interesting field for investigation, as these are attributed by some to the preexisting disease and not to the changes which follow the removal of diseased organs. This will, however, long continue a subject for discussion, as evidence is based upon these changes taking place in women suffering from disease capable of producing nervous manifestations, and the lower animals with inferior nervous organization would not respond to experimental research on such lines. True conservative gynecology often means the complete removal of diseased tissues when this conduces to the health and comfort of the patient or the preservation of as much of the tissue as will conserve the greatest degree of health and comfort to the patient.

